# Special Issue “Neurodegenerative Diseases: Molecular Mechanisms and Therapies, 3rd Edition”

**DOI:** 10.3390/ijms27041980

**Published:** 2026-02-19

**Authors:** Zhidong Zhou, Alexandre Hiroaki Kihara

**Affiliations:** 1 National Neuroscience Institute of Singapore, 11 Jalan Tan Tock Seng, Singapore 308433, Singapore; 2 Signature Research Program in Neuroscience and Behavioral Disorders, Duke-NUS Graduate Medical School Singapore, 8 College Road, Singapore 169857, Singapore; 3 Neurogenetics Laboratory, Universidade Federal do ABC, São Bernardo do Campo 09606-045, SP, Brazil; 4 Center for Mathematics, Computing and Cognition, Universidade Federal do ABC, São Bernardo do Campo 09606-045, SP, Brazil

## 1. Neuroinflammation as a Convergent Driver and Therapeutic Target

Neuroinflammation has emerged as a unifying pathological feature across neurodegenerative diseases, acting both as an initiator and amplifier of neuronal injury [1,2]. Several articles in this Special Issue directly address inflammatory mechanisms and their modulation [3,4,5]. Wu and colleagues present a systematic review and meta-analysis examining the therapeutic potential of cannabidiol (CBD) in Alzheimer’s disease-related neuroinflammation [3]. By synthesizing evidence from preclinical models and early-stage clinical studies, the authors demonstrate that CBD consistently reduces inflammatory markers such as GFAP, IL-6, and iNOS, particularly in animal models. Importantly, the review highlights CBD’s favorable safety profile and modest behavioral benefits in humans, while also underscoring the need for larger, biomarker-driven clinical trials [3]. This situates CBD within a growing class of immunomodulatory compounds that may complement conventional amyloid- or tau-focused strategies in AD.

Neuroinflammatory processes are also examined in the context of PSP by Chunowski et al., who investigate peripheral inflammatory markers and their relationship to cognitive function in PSP-RS and PSP-P [4]. Their findings reveal negative correlations between cognitive performance and systemic inflammation indices such as the neutrophil-to-lymphocyte ratio and platelet-to-lymphocyte ratio [4]. Intriguingly, interleukin-1β appears to exert a weak protective association, highlighting the complex and context-dependent roles of cytokines in neurodegeneration [4]. This study reinforces the concept that peripheral immune signatures can reflect central pathology and may serve as accessible biomarkers for disease monitoring.

Complementing these clinical observations, Barczuk and colleagues provide mechanistic insight into inflammation-associated neurotoxicity using an in vitro microglial model [5]. Their study demonstrates that noradrenaline protects human microglial cells from apoptosis and DNA damage induced by lipopolysaccharide and amyloid-β aggregates [5]. By modulating apoptotic signaling, oxidative stress, and HIF-1α expression, noradrenaline emerges as a potent regulator of microglial resilience [5]. These findings align with clinical observations of locus coeruleus degeneration in AD and suggest that restoring noradrenergic tone may have therapeutic relevance [5]. This work complements recent evidence that glial modulation, beyond amyloid-centric approaches, can alter disease trajectories, as seen in studies demonstrating that selective inhibition of microglial NLRP3 inflammasome attenuates amyloid pathology and cognitive decline [6] and that peripheral immune modulation impacts CNS inflammation [7]. The CBD review thus arrives at a critical inflection point: moving from classical anti-amyloid strategies toward immune-centric and glia-modulating therapy.

## 2. Mitochondrial Integrity, Oxidative Stress, and Cellular Resilience

Mitochondrial dysfunction and oxidative stress are central contributors to neuronal vulnerability, influencing both energy metabolism and regulated cell death pathways [8]. This theme is prominently addressed in the study by Eixarch et al., which examines the effects of cladribine on central nervous system cells in models relevant to multiple sclerosis [1]. Contrary to concerns about neurotoxicity, the authors demonstrate that therapeutic concentrations of cladribine preserve glial viability and function while enhancing neuronal resistance to oxidative stress [1]. These results provide important reassurance regarding the CNS safety of cladribine and suggest that its benefits may extend beyond immunomodulation to direct neuroprotection.

The vulnerability of astrocytes and their neuroprotective capacity are further explored by Rodríguez-Pozo and colleagues in a study of perinatal ethanol exposure in a triple-transgenic AD mouse model [2]. Their findings reveal that early-life ethanol exposure exacerbates astrogliosis, elevates pro-inflammatory markers, and disrupts endocannabinoid signaling pathways involving GPR55 and palmitoylethanolamide [2]. This work highlights how developmental insults can compromise glial-mediated neuroprotection and increase susceptibility to neurodegenerative processes later in life. Importantly, it identifies the GPR55/PEA axis as a potential target for restoring astrocytic homeostasis.

## 3. Neurotransmitter Imbalance and Network Compensation

Beyond inflammation and metabolism, alterations in neurotransmitter systems play a critical role in shaping disease trajectories [9]. Pellicano et al. investigate cerebellar glutamatergic and GABAergic systems in individuals with isolated REM sleep behavior disorder and de novo PD [6]. Using magnetic resonance spectroscopy, the authors reveal subtle but significant shifts in the excitatory–inhibitory balance, suggesting cerebellar hyperexcitability in early PD [6]. The inverse relationship between neurotransmitter ratios and neuropsychiatric symptoms in prodromal stages points to compensatory network mechanisms that may precede overt motor dysfunction [6]. This study underscores the cerebellum’s underappreciated role in PD pathophysiology and highlights neurochemical imaging as a promising tool for early detection.

This aligns with growing evidence that circuit-level alterations occur before classical symptom onset. Complementary studies using functional MRI have identified early cortico-striatal and thalamo-cortical connectivity changes in iRBD that predict progression to PD [10]. Together, these findings support the emerging view that PD pathology encompasses network reorganization and compensatory dynamics well before motor dysfunction.

## 4. Metabolism, Diet, and Peripheral Biomarkers

An emerging body of evidence suggests that systemic metabolism and dietary factors influence neurodegenerative disease risk and progression. Zhi Dong and colleagues contribute to this growing body of literature by analyzing plasma levels of food-derived metabolites in PD using data from the Parkinson’s Progression Markers Initiative [7]. Their study reveals significantly reduced levels of caffeine and related metabolites in PD patients, particularly among carriers of LRRK2 and GBA1 mutations [7]. The inverse correlation between metabolite levels and motor severity supports the notion that dietary or metabolic signatures may serve as accessible biomarkers and potential modifiers of disease progression [7]. This work provides a compelling rationale for integrating nutritional strategies into personalized PD management.

## 5. Molecular Diagnostics and Precision Neurology

Advances in biomarker technology are transforming the diagnostic landscape of neurodegenerative diseases. Yi, Tan, and Zhou provide a focused commentary on the α-synuclein seeding amplification assay (SAA), a highly sensitive method for detecting misfolded α-synuclein species [8]. The authors discuss the prion-like principles underlying the assay, its applicability across sporadic and genetic forms of PD, and its potential utility in blood-based diagnostics [8]. While acknowledging current limitations related to standardization and specificity, the commentary positions SAA as a cornerstone technology for early diagnosis, patient stratification, and therapeutic monitoring in precision neurology [8]. The broader field has rapidly validated SAA across large cohorts, with high specificity distinguishing PD from controls and other movement disorders [11]. These assays are now being investigated as inclusion tools in clinical trials, reflecting a major advance in molecular diagnostics for synucleinopathies.

## 6. Epigenetic Plasticity and Lifelong Interventions

The final to this Special Issue broadens the perspective by addressing epigenetic regulation as a modifiable determinant of brain health. Kukla-Bartoszek and Głombik review evidence showing that physical exercise induces lasting epigenetic modifications that enhance neuroplasticity, cognitive function, and emotional regulation across the lifespan [9]. By integrating data on DNA methylation, histone modifications, and non-coding RNAs, the authors argue that exercise acts as a powerful, non-pharmacological intervention capable of counteracting aging and disease-related decline [9]. This review reinforces the importance of lifestyle factors in neurodegeneration and highlights epigenetic mechanisms as a bridge between environment and long-term brain resilience.

## 7. Concluding Perspectives

Collectively, the contributions in the current Special Issue reflect a paradigm shift in neurodegenerative disease research, from reductionist models toward integrated, network-based understanding. They emphasize that neuronal survival and degeneration are governed by the interplay among immune responses, metabolic state, neurotransmitter balance, and epigenetic regulation, shaped across developmental and aging trajectories. Importantly, these contributions span multiple disease entities, revealing shared mechanisms that transcend traditional diagnostic boundaries.
